# CTRP13 attenuates the expression of LN and CAV-1 Induced by high glucose via CaMKKβ/AMPK pathway in rLSECs

**DOI:** 10.18632/aging.103234

**Published:** 2020-06-17

**Authors:** Qi Zhang, Xiang’e Niu, Limin Tian, Jing Liu, Ruilan Niu, Jinxing Quan, Jing Yu, Wenyan Lin, Zibing Qian, Peiyun Zeng

**Affiliations:** 1 Department of Endocrinology, Gansu Provincial Hospital, Lanzhou 730000, Gansu Province, China; 2 Clinical Research Center for Metabolic Disease, Lanzhou 730000, Gansu Province, China; 3 School of Clinical Medicine, Gansu University of Chinese Medicine, Lanzhou 730000, Gansu Province, China; 4 School of Life Sciences, Lanzhou University, Lanzhou 730000, Gansu Province, China

**Keywords:** CTRP13, laminin, AMPK, high glucose, LSECs

## Abstract

Objective: To investigate the effect and mechanism of CTRP13 on hepatic sinusoidal capillarization induced by high glucose in rat liver sinusoidal endothelial cells (rLSECs).

Results: CTRP13 was reduced in high glucose-treated rLSECs. High glucose increased LN and CAV-1 expression and inhibited CaMKKβ and AMPK phosphorylation. CTRP13 overexpression protected rLSECs against high glucose-induced increase of LN and CAV-1 expression. Moreover, CTRP13 overexpression increased high glucose-induced inhibition of CaMKKβ and AMPK activation in CTRP13-overexpressing rLSECs. Inhibition of CaMKKβ and AMPK disturbed the protective effects of CTRP13 in high glucose-induced increase of LN and CAV-1. Hepatic steatosis was enhanced and basement membrane was thickened in liver of diabetic fatty liver rats.

Conclusions: Our data identified the protective role of CTRP13 in hepatic sinusoidal capillarization induced by high glucose via activating CAMKKβ/AMPK pathway. CTRP13 may be a potential target for screening and treating diabetic fatty liver.

Methods: Construct lentiviral CTRP13 overexpression vector and transfect rLSECs. Use STO-609 (a CaMKKβ inhibitor) or Compound C (an AMPK inhibitor) to treat rLSECs. CTRP13, CaMKKβ, AMPK, laminin (LN) and caveolin-1 (CAV-1) were detected by qRT-PCR and Western blotting. Establish rat model of diabetic fatty liver. Use immunohistochemistry, hematoxylin-eosin and silver staining to observe the histopathological features of liver.

## INTRODUCTION

Type 2 diabetes mellitus (T2DM) is an independent risk factor for the formation and development of non-alcoholic fatty liver disease (NAFLD) [1], potentially complicated by hepatic microangiopathy and liver inflammation [2]. It is reported that more than 70% of patients with T2DM have NAFLD [3, 4]. Considering the large number of T2DM patients worldwide [5], the burden of NAFLD seems to be enormous [6, 7]. However, the specific pathogenesis is still unclear.

NAFLD is associated with hepatic sinusoidal capillarization [8]. Capillarization, is that liver sinusoidal endothelial cells (LSECs) lack fenestration and develop an organized basement membrane [9]. Study has indicated that LSECs changes favour steatosis development and set the stage for NAFLD progression [9]. Previous studies demonstrate that hepatic sinusoidal capillarization undergo increased laminin (LN) and caveolin-1 (CAV-1) expression [10]. However, the exact mechanism responsible for hepatic sinusoidal capillarization induced by T2DM remains to be elucidated [11].

C1q/tumor necrosis factor-related protein 13 (CTRP13) is a novel adipokine involved in regulating lipid and glucose metabolism [12]. Research on members of the CTRPs family to address metabolic disorders including metabolic syndrome, obesity, and diabetes, has drawn great attention [13, 14]. Multiple lines of evidence have proven that CTRP13 is clearly decreased in patients with T2DM and NAFLD [15, 16]. Our previous studies have shown that serum levels of CTRP13 are reduced in T2DM combined with NAFLD patients. We speculate that CTRP13 may play an important role in the regulation of fatty liver development. However, none of the studies have assessed how CTRP13 plays an important role in fatty liver formation.

AMP-activated protein kinase (AMPK), a conserved serine/threonine kinase, is a critical sensor of the regulation of cellular energy homeostasis and metabolic pathways [17]. Some studies demonstrate that CTRP13 can enhance fatty acid oxidation [18] and promote glucose uptake in adipocytes, myotubes and hepatocytes via activating the AMPK signaling pathway [12, 19]. Calcium/calmodulin-dependent protein kinase kinase beta (CaMKKβ), as one of the upstream kinases of AMPK, can regulate AMPK activation, and activation of CaMKKβ is beneficial for microglia/macrophage anti-inflammatory activation [20, 21]. However, it is unclear whether CaMKKβ/AMPK pathway is regulated by CTRP13 to participate in the formation and development of hepatic sinusoidal capillarization.

Based on the above, CTRP13 may play a central role in the regulation of sinusoidal capillarization. In this study, we explored the regulatory effect of CTRP13 on hepatic sinusoidal capillarization induced by high glucose from in vitro and in vivo models. It is found that high glucose inhibits CTRP13 expression, and CTRP13 protects against high glucose-induced hepatic sinusoidal capillarization by activating CaMKKβ/AMPK pathway.

## RESULTS

### Obtained rLSECs express CD31 in culture

**Figure 1:**
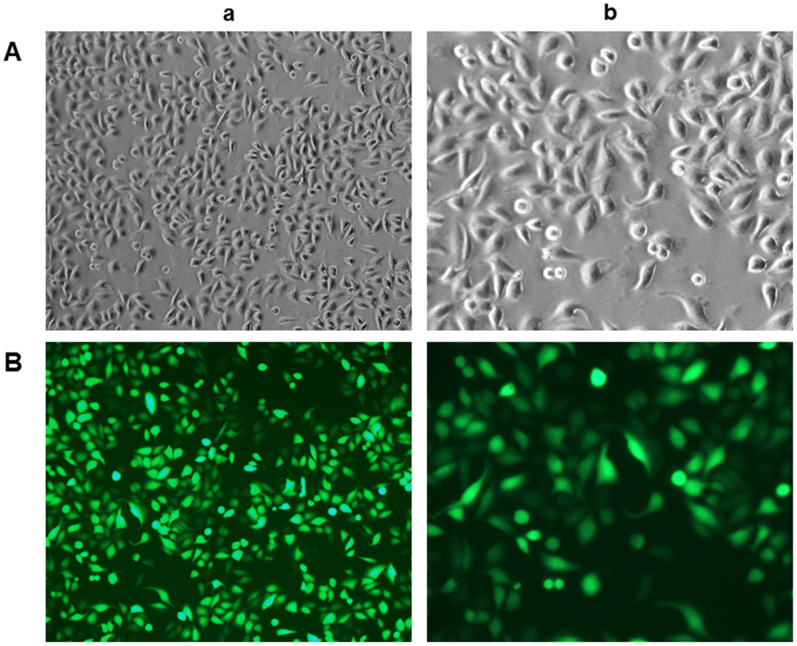
**Characterisation and identification of rat liver sinusoidal endothelial cells (rLSECs) in vitro.** (**A**) The morphology of rLSECs under white light. The cells grew by static adherence and the morphology of rLSECs were polygon or fusiform, resembling cobblestones under inverted microscopy. (**B**) Representative images of immunofluorescence staining of CD31 in rLSECs. Cells were stained with anti-CD31 antibodies. CD31 is uniformly expressed in rLSECs. Fluorescence images were acquired at an original magnification (green, CD31 expression). (a. ×100; b. ×200)

To confirm the identity of rLSECs, we assessed the expression of CD31 in rLSECs using immunofluorescence staining after culturing the cells for 48 h. The primary rLSECs were polygonal or fusiform with a cobblestone appearance, as demonstrated by inverted microscopy under white light in culture (Figure 1A). We found that CD31 was expressed on the surface of rLSECs, as indicated by green fluorescence under an inverted fluorescence microscope (Figure 1B).

### Effect of high glucose on CTRP13, CaMKKβ, AMPK, LN and CAV-1 expression in rLSECs

**Figure 2:**
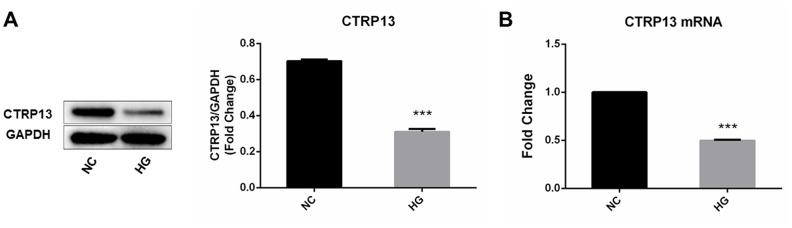
**The effect of high glucose on CTRP13 expression in rLSECs. rLSECs were treated with high glucose (25 mM) for 24 h.** (**A**) Western blotting showing the expression levels of CTRP13 in the rLSECs treated by high glucose; (**B**) The mRNA expression levels of CTRP13 were detected using qRT-PCR analysis. The results were normalised to GAPDH mRNA levels. All data are representative as mean ± S.D. from three independent experiments. ***P < 0.001 vs. control group.

**Figure 3:**
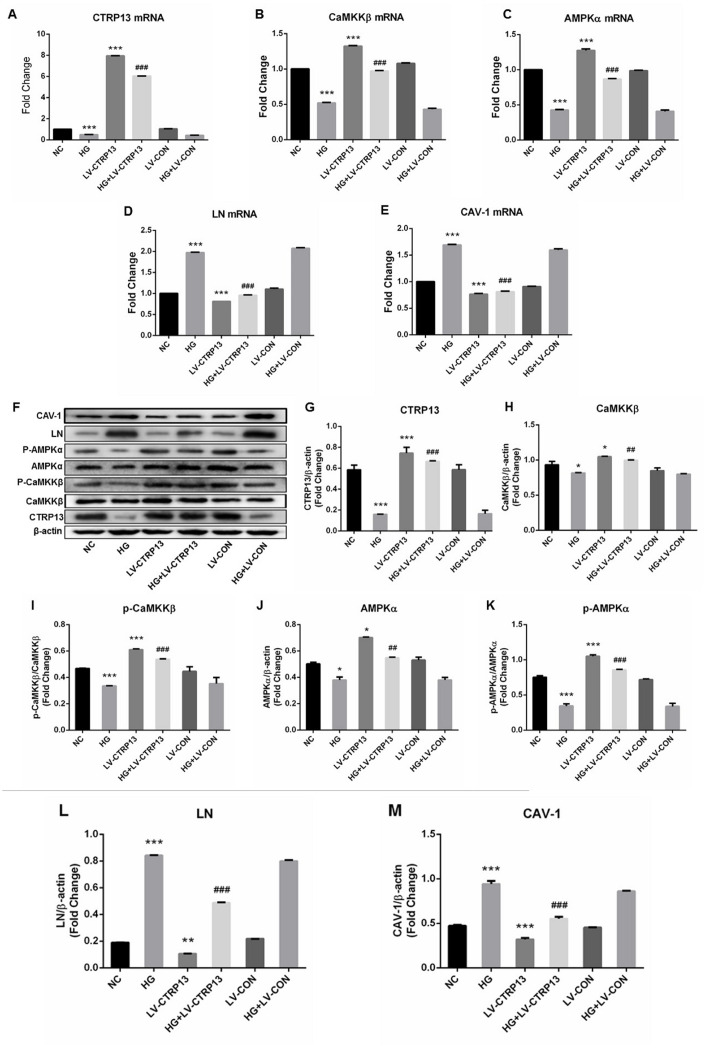
**The effect of high glucose on the expression levels of CTRP13, p-CaMKKβ, CaMKKβ, p-AMPK, AMPK, LN and CAV-1 in rLSECs transfected by recombinant LV-CTRP13.** (**A**) qRT-PCR analysis of CTRP13 mRNA. (**B**) qRT-PCR analysis of CaMKKβ mRNA. (**C**) qRT-PCR analysis of AMPKα mRNA. (**D**) qRT-PCR analysis of LN mRNA. (**E**) qRT-PCR analysis of CAV-1 mRNA. (**A**–**E**) The results were normalised to β-actin mRNA levels. (**F**) The protein expression levels of each group were detected using western blotting, and β-actin was used as a loading control. (**G**) Western blotting results showing relative CTRP13 expression. (**H**) Western blotting results showing relative CaMKKβ expression. (**I**) Western blotting results showing relative phos-CaMKKβ expression of CaMKKβ activation. (**J**) Western blotting results showing relative AMPKα expression. (**K**) Western blotting results showing relative phos-AMPKα expression of AMPKα activation. (**L**) Western blotting results showing relative LN expression. (**M**) Western blotting results showing relative CAV-1 expression. (**F**–**M**) β-actin (42 kDa) represents the loading control. All results are expressed as mean±S.D. from three independent experiments, ^*^P < 0.05, ^**^P < 0.01, ^***^P < 0.001 vs. control. ^*##*^*P* < 0.01, ^*###*^*P* < 0.001 vs the high glucose + LV-CON group, respectively.

The effect of high glucose on rLSECs has been reported in our previous research [22]. To investigate the effect of high glucose on CTRP13, CaMKKβ, AMPK, LN and CAV-1 expression in rLSECs, we measured the expression level of CTRP13, CaMKKβ, AMPK, LN and CAV-1 using qRT-PCR and Western blot analyses. Firstly, the CTRP13 expression levels were examined in response to high glucose. Real-time qPCR and Western blot analyses revealed that incubation with 25 mM high glucose (HG) for 24 hours resulted in a decrease of CTRP13 expression in rLSECs (Figure 2). Besides, results showed that high glucose resulted in a decrease of p-CaMKKβ and p-AMPK expression and an increase of LN and CAV-1 expression in rLSECs (Figure 3B–3F and 3H–3M). Hence, our results substantiate that high glucose reduces CTRP13, p-CaMKKβ and p-AMPK expression and increases LN and CAV-1 formation in rLSECs.

### Lentiviral CTRP13 overexpression vector (LV-CTRP13) successfully increased the expression of CTRP13 in rLSECs

**Figure 4:**
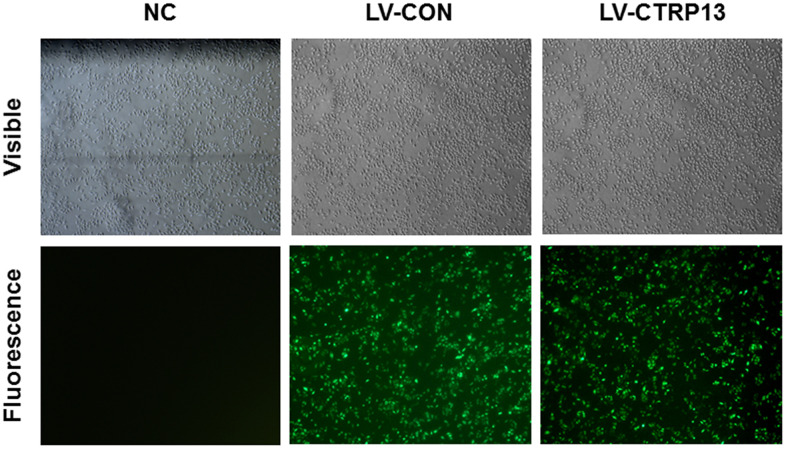
**Lentiviral transfection of rLSECs.** The infection rate of LV-CTRP13 and LV-CON in the rLSECs at an MOI of 100 were observed under a fluorescence microscope at 72 h following infection, respectively (×40).

In order to examine the role of CTRP13 in high glucose-induced increase of LN and CAV-1 expression, rLSECs were infected with LV-CTRP13 or LV-CON. After 96 hours, the fluorescence images showed that green fluorescence intensity was clearly increased in rLSECs transfected with LV-CTTRP13 and infection efficiency was ~90% (Figure 4). In addition, we quantified the expression of CTRP13 mRNA and protein in LV-CTRP13-treated rLSECs and control group cells using qRT-PCR (Figure 3A) and western blotting (Figure 3F and 3G). Results revealed that the CTRP13 expression was significantly increased in rLSECs transfected with LV-CTRP13 compared to the control group by 11-fold (Figure 3A).

### CTRP13 overexpression inhibited high glucose-induced increase of LN and CAV-1 expression in rLSECs

Cells (infected with either LV-CTRP13 or LV-CON) were stimulated with 25 mM high glucose. The expression changes of LN and CAV-1 in CTRP13-overexpressing cells followed by treatment with high glucose (HG) for 24 hours were examined. The results demonstrated that CTRP13 overexpression attenuated HG-induced increase of LN and CAV-1 expression at both mRNA and protein levels. The mRNA levels of LN (Figure 3D) and CAV-1 (Figure 3E) were reduced by 51.6% and 51.9% and the protein levels of LN (Figure 3F, 3L) and CAV-1 (Figure 3F, 3M) were reduced by 41.8% and 31%, respectively. All these results indicate that high glucose promotes LN and CAV-1 expression in rLSECs, at least in part, by the inhibition of CTRP13 expression.

### CTRP13 overexpression increased HG-induced inhibition of p-CaMKKβ and p-AMPK activation in rLSECs

To explore the molecular mechanisms by which CTRP13 overexpression inhibited HG-induced increase of LN and CAV-1 expression, we detected the expression levels of CaMKKβ and AMPK in rLSECs. AMPK activation is correlated with the phosphorylation state at threonine (Thr)-172 on the AMPK a subunit. Our data revealed that treatment with LV-CTRP13 effectively restored HG-induced inhibition of p-CaMKKβ Ser511 and p-AMPKα Thr172 levels in rLSECs (Figure 3B, 3C, 3F and 3H–3K). The protein levels of p-CaMKKβ (Figure 3F, 3I) and p-AMPK (Figure 3F, 3K) were increased by 76.3% and 74.6% in LV-CTRP13 cells compared with the LV-CON under high glucose. Figure 3F showed the total CaMKKβ and AMPK protein levels of the Western blot grayscale band in different groups seemed to have little difference. However, statistical analysis showed that changes of total CaMKKβ and AMPK protein levels were consistent with changes of CaMKKβ and AMPK mRNA levels in different groups, and the differences between the groups were statistically significant. These results indicate that, in addition to the increase in the level of CTRP13, caused by transfection, the expression levels of p-CaMKKβ and p-AMPK were also higher in LV-CTRP13-transfected cells when compared to the LV-CON.

### Impact of STO-609 and compound C treatment on rLSECs viability

**Figure 5:**
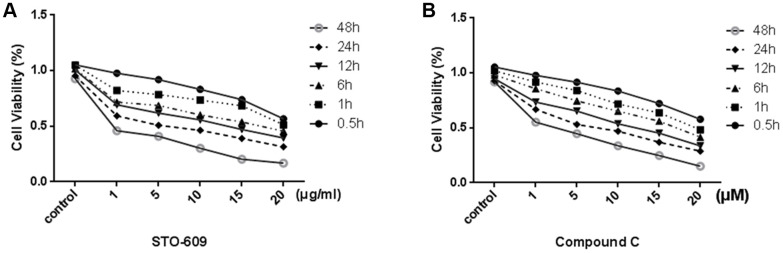
**Effect of STO-609 and Compound C on Cell Viability of rLSECs.** rLSECs were treated with STO-609 (0, 1, 5, 10, 15 and 20 μg/ml) and Compound C (0, 1, 5, 10, 15 and 20 μM) for 0.5, 1, 6, 12, 24 or 48 h, respectively. Cell viability was determined by MTT test. All data is expressed as mean ± SD. *P*<0.05 versus control. (**A**) The effect of STO-609 on rLSEC viability (control is untreated group). (**B**) The effect of STO-609 on rLSEC viability (control is untreated group). All data is expressed as mean ± SD. from three independent experiments. *P* < 0.05 versus control.

In order to test the effective treatment concentrations of STO-609 and Compound C, cells are respectively treated with 0, 1, 5, 10,15 or 20 mg/ml STO-609 and 0, 1, 5, 10,15 or 20 μM Compound C for 0.5, 1, 6, 12, 24 or 48 h and then assessed using the MTT assay. We observed that the cells viability decreased in a dose- and time-dependent manner (*p* < 0.05; Figure 5). The concentrations of STO-609 >10 mg/ml and Compound C >10 μM had noticeable cytotoxic and inhibitory effects. Furthermore, exposure to 20 mg/ml STO-609 and 20 μM Compound C for 48 h decreased significantly the viability of rLSECs (by 76% and 76.8% total from control, respectively). Therefore, we selected the 10 mg/ml STO-609 (55.1%) and 10 μM compound C (54.2%) for 24h to be used in the following study carried out in rLSECs, respectively.

### CTRP13 overexpression inhibited HG-induced LN and CAV-1 expression through AMPK signaling in rLSECs

**Figure 6:**
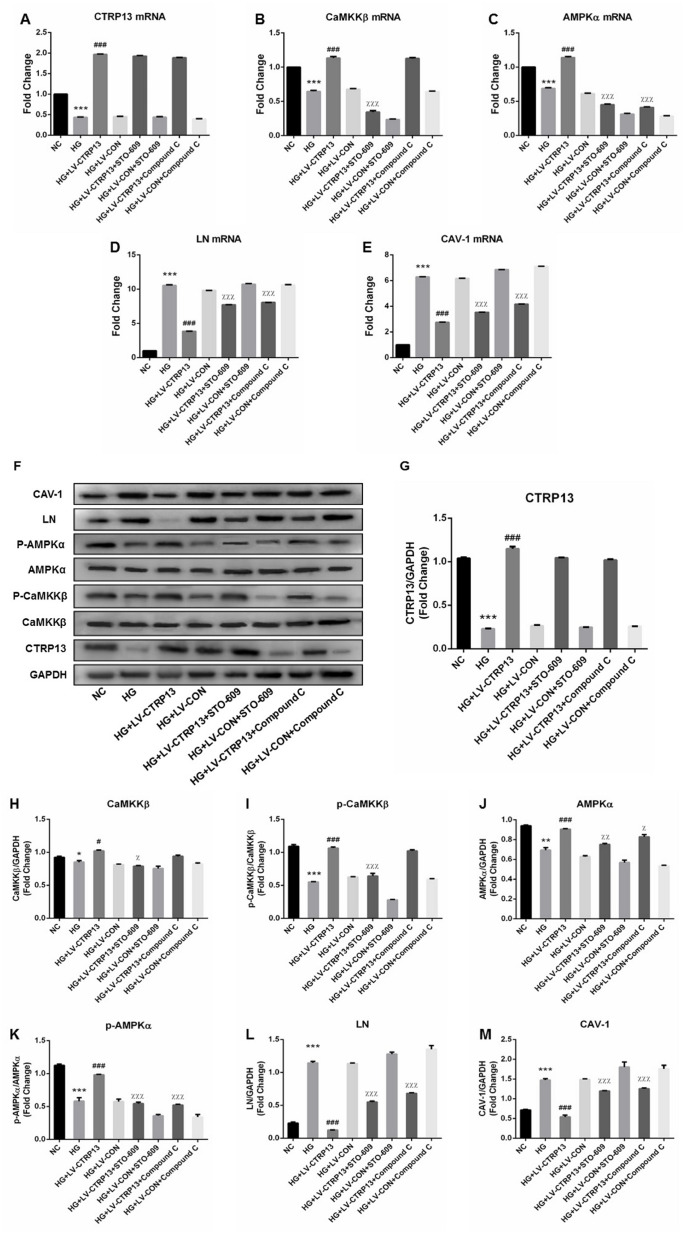
**Overexpression of CTRP13 elevated the activation of CaMKKβ/AMPK pathway and inhibited the expression of LN and CAV-1 in rLSECs.** Cells were transfected with LV-CTRP13 or LV-CON and blocked with CaMKK or AMPK inhibitor. All samples were treated with 25 mg/ml high glucose for 24 h. Untreated intact samples were run as a control in each experiment. (**A**) qRT-PCR analysis of CTRP13 mRNA. (**B**) qRT-PCR analysis of CaMKKβ mRNA. (**C**) qRT-PCR analysis of AMPKα mRNA. (**D**) qRT-PCR analysis of LN mRNA. (**E**) qRT-PCR analysis of CAV-1 mRNA. The results were normalised to GAPDH mRNA levels. (**F**) The protein expression levels of each group were detected using western blotting, and GAPDH was used as a loading control. (**G**) Western blotting results showing relative CTRP13 expression. (**H**) Western blotting results showing relative CaMKKβ expression. (**I**) Western blotting results showing relative p-CaMKKβ expression of CaMKKβ activation. (**J**) Western blotting results showing relative AMPKα expression. (**K**) Western blotting results showing relative p-AMPKα expression of AMPKα activation. (**L**) Western blotting results showing relative LN expression. (**M**) Western blotting results showing relative CAV-1 expression. GAPDH (37 kDa) was used as a loading control. All results are expressed as mean**±**S.D. from three independent experiments, ^*^P < 0.05, ^**^P < 0.01, ^***^P < 0.001vs. control. ^*#*^*P* < 0.05, ^*###*^*P* < 0.001 vs the high glucose + LV-CON group. ^χ^*P* < 0.05, ^χχ^*P* < 0.01, ^χχχ^*P* < 0.001 vs the high glucose + LV-CTRP13 group, respectively.

In the way of reinforcing our results, same as Figure 3, Figure 6 further confirmed that the expressions of CTRP13, p-CaMKKβ and p-AMPK were decreased and the expressions of LN and CAV-1 were increased in rLSECs treated with high glucose. In addition, treatment with LV-CTRP13 effectively rescued HG-induced inhibition of p-CaMKKβ and p-AMPK activation and decreased HG-induced increases of LN and CAV-1 in rLSECs compared to the LV-CON (Figure 6). In order to explore the molecular mechanisms by which CTRP13 inhibited HG-induced increase of LN and CAV-1 expression in rLSECs, we detected the levels of AMPK signaling. Compound C, a specific inhibitor of AMPK, significantly increased LV-CTRP13-induced decrease of LN and CAV-1 expression in rLSECs (Figure 6D–6F, 6L and 6M). Compound C had no effect on the expressions of CTRP13 and the phosphorylation levels of CaMKKβ (Figure 6A, 6B and 6F–6I). Taken together, these results confirm that CTRP13 overexpression inhibits HG-induced increase of LN and CAV-1 expression in rLSECs transfected with LV-CTRP13 via activating AMPK signaling pathway.

### LV-CTRP13 induced AMPK signaling pathway in HG-treated rLSECs by enhancing CaMKKβ activation

In order to further examine the role of CaMKKβ signaling (the upstream kinases of AMPK) in LV-CTRP13-induced decrease of LN and CAV-1 expression, rLSECs were pretreated with CaMKKβ inhibitor (STO-609, 10 mg/ml) for 24 h followed by transfected with LV-CTRP13. STO-609 significantly increased the LV-CTRP13-induced decrease of LN and CAV-1 expression in rLSECs (Figure 6D–6F, 6L and 6M). Consistent with the inhibition of CaMKKβ activity, STO-609 significantly reduced LV-CTRP13-mediated phosphorylation of AMPKα at Thr172 in rLSECs transfected with LV-CTRP13 (Figure 6C, 6F, 6I and 6J). These results suggest that the activation of CaMKKβ is involved in the LV-CTRP13-induced phosphorylation of AMPK in rLSECs transfected with LV-CTRP13. Meanwhile, we found that the levels of changes in CaMKKβ phosphorylation and AMPK phosphorylation were higher than the levels of differences between total CaMKK and AMPK, implying the possibility that any changes the impact in this pathway by CTRP13 was largely due to altered phosphorylation in CTRP13 overexpression cells. In conclusion, high glucose promoted expression of LN and CAV-1 by inhibiting CaMKKβ/AMPK signaling, and the overexpression of CTRP13 inhibited HG-induced increase of LN and CAV-1 expression by activating CaMKKβ/AMPK signaling activation.

### Liver histopathological features of diabetic fatty liver rats

**Figure 7:**
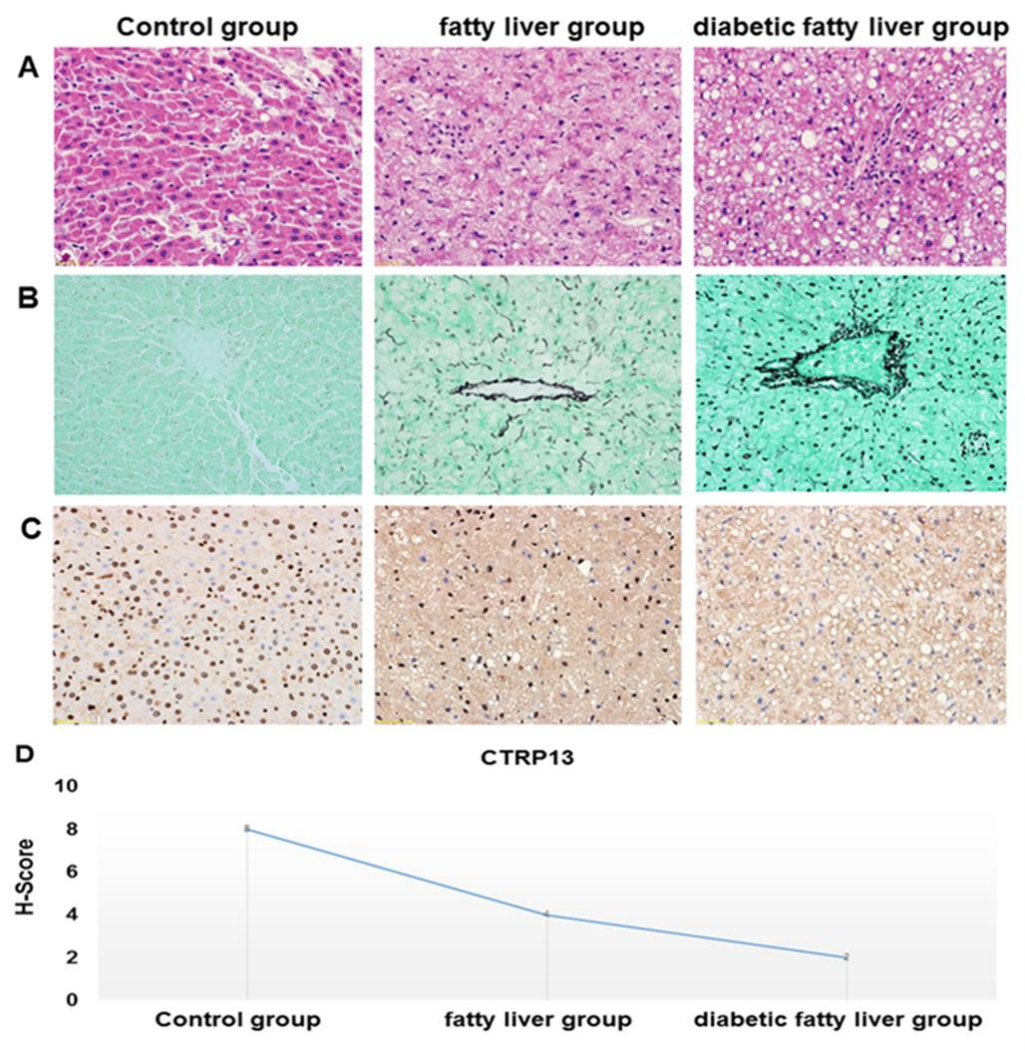
**Histopathological features of the liver tissues.** (**A**) Representative images of HE staining in livers from different groups of rats (× 40). HE staining was utilized to analyze histological abnormalities. (**B**) Representative images of the gomori methenaminutese silver staining in liver sections from different groups of rats (×40). (**C**) Representative images of immunohistochemistry staining of CTRP13 in livers from different groups of rats (× 40). (**D**) Quantification of CTRP13-positive cells in liver sections from different groups of rats.

HE staining of liver sections showed that hepatocytes were arranged in a regular pattern to form a liver plate, the morphology and structure were normal under the optical microscope in the control rats. However, in livers from fatty liver rats and diabetic fatty liver rats, the hepatocytes were loose in structure and disordered in arrangement, intracellular lipid accumulation and steatosis, the inflammation of liver aggravation (magnification, 400×). In liver tissues of diabetic fatty liver rats, steatosis was relatively more serious than the fatty liver rats, indicating that the rat model of diabetic fatty liver was successfully modeled (Figure 7A).

### Liver basement membrane changes of diabetic fatty liver rats

Gomori methenamine silver staining showed that reticular fibers are uniformly distributed, no thickening, fusion and collapse in liver of control rats. Compared with control rats, basement membrane and sinus space had more obvious and darker brown coloration, and reticular fibers lost normal tissue distribution and collapsed, merged, thickened, and entangled in the portal vein region in the liver of diabetic fatty liver rats (Figure 7B).

### Expression of CTRP13 in liver from diabetic fatty liver rats

In immunohistochemistry staining, the positive areas are dyed in blue. The positive areas of CTRP13 were shown a significantly decreasing trend in the liver from the control rats to fatty liver rats and diabetic fatty liver rats (Figure 7C and 7D), which clearly indicated that the expression of CTRP13 was reduced in the liver from diabetic fatty liver rats.

## DISCUSSION

NAFLD is associated with hepatic microangiopathy and inflammation caused by T2DM. Hepatic sinusoid endothelial dysfunction is an early event implicated in the progression to NAFLD [10]. LSECs line the capillaries of the microvasculature and possess fenestrae to facilitate filtration between the liver parenchyma and sinusoids [23], including lipoprotein metabolism [24]. A number of studies have suggested the involvement of LSECs dysfunction in the formation and development of NAFLD combined with T2DM [25, 26].

CTRPs are implicated in the pathophysiology of metabolic disorders in recent studies, such as NAFLD and T2DM. The serum level of CTRP13 was negatively correlated with fasting blood glucose in humans [15]. Our previous studies have shown that serum levels of CTRP13 were reduced in patients with T2DM combined with NAFLD. In this study, using the rat models of diabetic fatty liver, we found that CTRP13 was reduced in livers from diabetic fatty liver rats by immunohistochemistry staining (Figure 7C and 7D). Consistently, in vitro, the results of qRT-PCR and western blotting showed that CTRP13 was down-regulated in rLSECs treated by high glucose (Figure 2). These results for the first time demonstrate that CTRP13 is down-regulated under high glucose conditions in vivo and in vitro. Studies found that CTRP13 improves fatty acid-induced insulin resistance by suppressing lipid-induced stress signaling [12]. These findings that were reported earlier as well as our current findings are consistent with our long-standing speculation that CTRP13 plays an essential role in the modulation of diabetic fatty liver disease.

Normal LSECs have large amounts of open fenestrae and lack a continuous basement membrane [27]. These characteristics related to biochemical and metabolic adaptive mechanisms play an important role in alleviating progression of NAFLD [9]. Sinusoidal capillarization is characterized by the formation of basement membrane and defenestration of LSECs [28, 29]. Our histopathological analysis displayed, in liver of diabetic fatty liver rats, that the hepatocytes were loose in structure and disordered in arrangement and most hepatocytes showed steatosis, with a large number of lipid droplets in some cytoplasm (Figure 7A). The silver staining showed that liver from diabetic fatty liver rats had more obvious and darker brown coloration in basement membrane and sinus space (Figure 7B). Our data showed that diabetic fatty liver rats increased deposition of lipid droplets and the formation of basement membranes in liver tissues.

LN, a major component of basement membrane, rarely expressed in normal LSECs but gradually increased in the process of hepatic steatosis [30]. LN and type IV collagen deposition resulted in continuous basement membrane formation in LSECs. During hepatic fibrogenesis, LN is shown to be significantly increased [31], resulting in the formation of basement membrane [32]. LN plays a crucial role in regulating the function of LSECs in sinusoidal reconstruction. We found that high glucose increased LN expression in rLSECs, demonstrating that high glucose could result in continuous basement membrane formation of hepatic sinusoids. To further investigate the effect of CTRP13 on LN expression, we designed a LV-CTRP13 vector. We found that overexpression of CTRP13 down-regulated the expression levels of LN protein and inhibits the expression of LN mRNA in rLSECs transfected with LV-CTRP13 (Figure 3D, 3F and 3L). This demonstrates that CTRP13 contributes to the reversal of pathological changes such as the basement membrane formation of hepatic sinusoids induced by high glucose via down-regulating LN expression.

CAV-1 is a membrane protein involved in the maintenance of fenestrae in LSECs [33]. Consistent with the previous study, we found that CAV-1 was expressed in isolated rLSECs. It is confirmed that CAV-1 could regulate endothelial capillary-like tubular formation [34], and the contraction and dilatation of the fenestrae in LSECs [35, 36]. In other words, the changes and migration of CAV-1 might influence LSECs phenotype. Our results show that CAV-1 is up-regulated in rLSECs treated by high glucose, which may be the cause of fenestra contraction and defenestration [37]. However, CTRP13 overexpression inhibited HG-induced increase of CAV-1 expression in rLSECs transfected with LV-CTRP13 (Figure 3E, 3F and 3M). These results support the conclusion that CTRP13 down-regulation promotes the defenestration of rLSECs. Combined with the data above, we found that LN and CAV-1 were significantly up-regulated in rLSECs treated by high glucose, eventually promoting capillarization of hepatic sinusoids. The overexpression of CTRP13 markedly reversed HG-induced increase of LN and CAV-1 expression in the cells transfected with LV-CTRP13. The present study contributed to support the notion that HG-induced upregulation of LN and CAV-1 expression in rLSECs may depend, at least partially, on the downregulation of CTRP13. However, CTRP13 overexpression can ameliorate conformation of sinusoidal capillarization induced by high glucose through downregulating the expression levels of LN and CAV-1 in rLSECs.

The AMPK signaling pathway can inhibit gluconeogenesis through phosphorylation [38]. The AMPK pathway is closely related to metabolic diseases [39]. Previously, it has been shown that AMPK activators can markedly reduce inflammation and inhibit tissue inflammatory damage in various models [40, 41]. The present result shows that AMPK phosphorylation is down-regulated in rLSECs treated by high glucose. Several downstream mediators of CTRP13 signaling have been demonstrated in several different cell types. In the liver, adipose and skeletal muscle, CTRP13 is known to activate AMPK. CTRP13 suppresses gluconeogenesis in hepatocytes and promote glucose uptake in myotubes and adipocytes via activation of the AMPK signaling pathway [13]. These studies show that CTRP13 regulates various physiological processes by the activation of AMPK signaling pathway. Here, the results showed that treatment of rLSECs with high glucose decreased the expression of AMPK phosphorylation, while CTRP13 overexpression upregulated HG-induced downregulation of AMPK phosphorylation in rLSECs transfected with LV-CTRP13 (Figure 3C, 3F, 3I and 3J). Furthermore, we found that Compound C increased LV-CTRP13-induced decrease of LN and CAV-1 expression in cells transfected with LV-CTRP13 (Figure 6D–6F, 6L and 6M), indicating that AMPK inhibitor can markedly promote formation of basement membrane and defenestration in rLSECs. Our results show that CTRP13 overexpression decreases HG-induced increase of LN and CAV-1 expression by activating the AMPK signaling pathway in rLSECs, which ultimately mitigates hepatic sinusoidal capillarization induced by high glucose.

CaMKKβ, as one of the upstream kinase of AMPK, formed a stable complex with AMPK and promoted AMPK activation [42]. We found that high glucose reduced the phosphorylation levels of CaMKKβ and CTRP13 overexpression increased the activity of CaMKKβ in rLSECs. Furthermore, it is demonstrated that CTRP13 overexpression upregulated HG-induced inhibition of CaMKKβ signaling activation. The results showed that CTRP13 may protect cells against HG-induced increase of LN and CAV-1 expression by increasing CaMKKβ activity. STO-609 down-regulated LV-CTRP13-induced the activation of AMPK phosphorylation to some extent in rLSECs treated with LV-CTRP13 (Figure 6C, 6F, 6J and 6K). The effect of Compound C on the expression of CaMKKβ was not obvious. In addition, STO-609 and Compound C had no effect on CTRP13, while these inhibitor increased LV-CTRP13-induced decrease of LN and CAV-1 expression levels in rLSECs transfected with LV-CTRP13 (Figure 6D–6F, 6L and 6M). Transfection with LV-CTRP13 also markedly reversed the influence of high glucose on CaMKKβ/AMPK signaling pathway, and decreased HG-induced increase of LN and CAV-1 expression. These results show that CTRP13 can successively pass by activating the CaMKKβ in the upstream, and the AMPK signaling pathway can decrease the expression of LN and CAV-1, and finally extenuate hepatic sinusoidal capillarization induced by high glucose. In short, these results exhibited that CTRP13 overexpression could inhibit HG-induced upregulation of LN and CAV-1 expression by the activation of CaMKKβ/AMPK signaling pathway.

In conclusion, we show that CTRP13 increases HG-induced inhibition of CaMKKβ/AMPK activation to protect rLSECs from HG-induced upregulation of LN and CAV-1 expression. Our study provides new insight into the pathological significance of NAFLD that it is associated with CTRP13 down-regulation in T2DM patients. CTRP13 may be a novel therapeutic target for treating diabetic fatty liver disease progression. Therefore, identifying the exact mechanism by which CTRP13 is impacting hepatic sinusoidal capillarization at the molecular level will provide greater benefits towards screening and treating diabetic fatty liver disease with CTRP13 down-regulation.

## MATERIALS AND METHODS

### Animals

Male Wistar rats, with an average body weight of 200 g (180-220 g), were purchased from Gansu University of Chinese Medicine Experimental Animal Center (Lanzhou, Gansu, China) and fed in the specific pathogen free (SPF) animal laboratory. All animals maintained under standard housing conditions (12 h alternating light–dark cycle and 25±0.5 °C, with a relative humidity of 50%). Rats were randomly divided into three groups after 1 week of normal feeding as described previously: control group (basic feed feeding, n = 10), fatty liver group (high-fat diet feeding, n = 15) and diabetic fatty liver group (high-fat diet + low-dose STZ intraperitoneal injection, n = 20) [43]. On the 8^th^ weekend, rats were injected intraperitoneally with 28 mg/kg/d streptozotocin (STZ) after gavaged in high fat diet. Rats in the control group and the fatty liver group were injected intraperitoneally with the same amount of citric acid buffer solution. After 72 hours, serum glucose levels were measured for 3 days. The random blood glucose levels were greater than 16.7 mmol/L and / or the fasting blood glucose levels were greater than 11.1 mmol/L verify that diabetic rats was successfully established. The histopathological HE staining of liver showed steatosis of most hepatocytes, indicating the successful establishment of the diabetic fatty liver rat models. All procedures were in accordance with the National Institutes of Health Guide for the Care and Use of Laboratory Animals.

### Reagents

Dulbecco's modifed Eagle's medium (DMEM) were purchased from HyClone Laboratories (Logan, UT, USA). The primer pairs for *GAPDH, CTRP13,* LN and CAV-1 were synthesized by TaKaRa Biotechnology Co., Ltd. (Dalian, China). Rabbit polyclonal antibodies to CTRP13, CaMKKβ, AMPK, LN, CAV-1, GAPDH and goat anti-rabbit HRP-conjugated secondary antibodies were purchased from Bioss Biotechnology Co., Ltd. (Beijing, China). P-CaMKKβ (Ser511) antibody and p-AMPK (Thr172) (40H9) rabbit mAb were purchased from Cell Signaling Technology (Inc., USA). All other chemicals were purchased from commercial suppliers.

### Liver histopathological measurement

After 20 weeks, the livers were isolated and soaked in 10% paraformaldehyde phosphate buffer solution. After 2 days, Fragments of liver tissue was prepared according to the standard paraffin method and dehydrated, passing through different concentration of alcohol and embedded sections in paraffin blocks. Hematoxylin-eosin (HE) staining and gomori methenaminutese silver stain were used to observe histopathological features of the liver tissue.

### Immunohistochemistry staining

Antibodies against CTRP13 (1:500 dilution) were used as primary antibodies, and biotinylated goat anti-rabbit immunoglobulin Gas secondary antibody (1:100 dilution). Images were captured with an Olympus inverted fluorescence microscope under ×400 magnification and obtained and analysed using Metamorph software (Molecular Devices).

### rLSECs isolation, culture and identification

After the rats were sacrificed, the liver was removed, rat liver sinusoidal endothelial cells (rLSECs) were extracted from liver obtained from rats of control group as method described in our previous publication [38]. rLSECs were cultured in DMEM supplemented with 18% fetal bovine serum (FBS), 1% Penicillin-Streptomycin, 1% vascular endothelial growth factor. Cells were grown in a humidified atmosphere with 95% air and 5% CO-2 at 37°C. When cells reached 80% confluency, rLSECs were identified using an anti-CD31 antibody (1:500, Abcam) via immunocytochemistry and characteristic morphology in culture. Cells were cultured in growth media containing 25mM glucose to represent the high glucose (HG) condition.

### 3-(4,5-cimethylthiazol-2-yl)-2,5-diphenyltetrazolium bromide (MTT) assay for cell viability

The potential toxic effect of inhibitor on rLSECs was assessed using MTT assay. rLSECs were treated with various concentrations of STO-609 (0, 1, 5, 10, 15 or 20 μg/ml), a CaMKKβ inhibitor, and Compound C (0, 1, 5, 10, 15 or 20 μM), an AMPK inhibitor, for 0.5, 1, 6, 12, 24 or 48 h, respectively. Briefly, Cell upernatants were removed at the indicated time points, and cells were washed with PBS and incubated with 10 μL MTT (5 mg/ml) in the medium. After 4 h, MTT was removed and the coloured formazan was dissolved in 100 μL DMSO. The optical density (OD) was measured at a wavelength of 490 nm using a Thermo Scientific Microplate Reader (Multiskan Spectrum, Thermo Labsystems, Philadelphia, PA, USA).

### Lentivirus construction

The mRNA sequences of rat CTRP13 were retrieved from GenBank (ID: NM_001109403**).** GV367 vector encoding enhanced green fluorescence protein (eGFP), was doubly digested with the restriction enzyme NheI and AgeI (Shanghai Genechem Co. Ltd., Shanghai, China) and was then combined with PCR fragments encoding CTRP13 (a top strand: 5^'^- GGGTC AATATGTAATTTTCAGTG-3'; a bottom strand: 5'-CGTCGCCGTCCAGCTCGACCAG-3'). The total vector system composed of GV367, pHelper 1.0 and pHelper 2.0 vector were co-transfected into 293T cell line according to the instructions of the manufacturer of Lipofectamine 2000 (Life Technologies, Carlsbad, CA, USA). We obtained recombinant lentivirus CTRP13 (LV-CTRP13) from supernatant 48 h after transfection and utilized the ELISA to count the virus titer. Similarly, the control lentiviral vector which only expressed eGFP (LV-CON) was produced as a negative control. The virus titer was 5.0×10^8^ TU/mL in LV-CTRP13.

### Lentivirus transfection

The rLSECs in a 10 cm dish at 80% confluency were transfected with lentiviral vectors overexpressing CTRP13 (LV-CTRP13) and their negative control (LV-CON). To achieve optimal gene, Enhanced Infection Solution (ENi.s.) in the presence of 50 μg/mL polybrene was used according to the manufacturer’s instructions. At 96 hours after infection, cell phenotype and characteristics were observed under an inverted fluorescence microscope. The infection efficiency was evaluated by real-time quantitative polymerase chain reaction (qRT-PCR) and Western blot analysis.

### Quantitative reverse transcription polymerase chain reaction (qRT-PCR)

Total RNA was extracted using TRIzol reagent (Invitrogen). The purity and concentration of RNA were determined by measuring the ratio of absorbance at 260/280 nm. The reverse transcription and qRT-PCR were performed using the FastKing RT Kit (With gDNase) (TIANGEN, Beijing, China) and the SuperReal Premix Plus (SYBR Green) kit as per the company’s protocol, using the respective RNA as the templates. Calculation of Ct (threshold cycle) values for the amplification curves were achieved after subtracting *GAPDH* values for normalization. USA). The primer sequences used for qRT-PCR are listed in Table 1. The reaction was started with denaturation at 95 °C for 15 min, and then the cDNA was amplified for 45 cycles with the denaturation at 95 °C for 10 s, annealing at 55 °C for 30 s and extension at 72 °C for 32 s. Each sample was amplified in triplicates on the LightCycler RT-PCR System (Roche 480, New York, USA).

**Table 1:** Primer sequences for qRT-PCR.

**Gene**	**Forward(5'-3')**	**Reserve(5'-3')**
GAPDH (NM_017008.3)	GGCACAGTCAAGGCTGAGAATG	ATGGTGGTGAAGACGCCAGTA
CTRP13 (NC_000068.7)	TGTTCCATCCCGGGTATCTACTTC	ACTGGCATAGTCGTAATTCTGGTCA
CaMKK2 (NM_031338.1)	CAGGCCCGGTTCTACTTCCA	CAGGCCCGGTTCTACTTCCA
AMPKα(NC_005104.4)	CAGCACCGGAGGTCATCTCA	GCACGTGCTCATCGTCGAA
LN (NC_000084.6)	ACGGAGGTGGAGCCTTGTAG	TGGGAAGGGAGCATTTGG
Cav-1 (NM_031556.3)	CGGGAACAGGGCAACATCTAC	CTTCTGGTTCCGCAATCACATC

### Protein preparation and western blot analysis

The cells were lysed in RIPA (Radio-Immunoprecipitation Assay) lysis buffer (Beyotime, Beijing, China) containing 1% protease and phosphatase inhibitor (Roche Diagnostics, Basel, Switzerland). The protein concentrations were quantitated using bicinchoninic acid (BCA) protein assay method (Thermo Fisher Scientific, Grand Island, NY, USA). Equal concentration of proteins was separated using sodium dodecyl sulfate-polyacrylamide gel electrophoresis (SDS-PAGE) and transferred onto polyvinylidene fluoride (PVDF) membranes. Membranes were blocked using proteins from non-fat dry milk and probed with specific antibodies for CTRP13 (1:1500), CaMKKβ (1:1000), p-CaMKKβ (1:1000), AMPKα (1:1000), p-AMPKα (1:1000), LN (1:600), CAV-1 (1:1000), β-actin (1:5000), and GAPDH (1:5000). Finally, the membrane was incubated in a solution containing Chemiluminescent substrate. Densitometric analysis was performed using the ImageJ program.

### Statistical analyses

The data presented are the means ± standard deviation (SD) from at least three independent experiments. The statistical significance between the groups were performed by one-way analysis of variance (ANOVA) followed by Tukey’s multiple comparisons test by SPSS 21.0 and GraphPad 6.0 software. Values of *P*<0.05 was considered as a statistically significant.
